# Generation of patient-derived models from a metastatic pediatric diffuse leptomeningeal glioneuronal tumor with *KIAA1549::BRAF* fusion

**DOI:** 10.1007/s00401-022-02473-w

**Published:** 2022-08-04

**Authors:** Julie Messiaen, Annelies Claeys, Aniket Shetty, Lien Spans, Marleen Derweduwe, Anne Uyttebroeck, Bart Depreitere, Isabelle Vanden Bempt, Raf Sciot, Keith L. Ligon, David T. W. Jones, Sandra A. Jacobs, Frederik De Smet

**Affiliations:** 1 Department of Pediatric Hematology and Oncology, University Hospitals Leuven, Louvain, Belgium; 2 Department of Imaging and Pathology, Translational Cell and Tissue Research, KU Leuven, Louvain, Belgium; 3 Dana Farber Cancer Institute, Department of Pathology, Boston, MA USA; 4 Department of Human Genetics, University Hospitals Leuven, Louvain, Belgium; 5 Department of Oncology, KU Leuven, Louvain, Belgium; 6 Department of Neurosurgery, University Hospitals Leuven, Louvain, Belgium; 7 Research Group Experimental Neurosurgery and Neuroanatomy, Department of Neurosciences, KU Leuven, Louvain, Belgium; 8 Department of Human Genetics, KU Leuven, Louvain, Belgium; 9 Department of Pathology, University Hospitals Leuven, Louvain, Belgium; 10 Hopp Children’s Cancer Center at the NCT Heidelberg (KiTZ), Heidelberg, Germany; 11 Division of Pediatric Glioma Research, German Cancer Consortium (DKTK), German Cancer Research Center (DKFZ), Heidelberg, Germany

Diffuse leptomeningeal glioneuronal tumors (DLGNTs) were recognized as a separate entity in the 2016 WHO classification update [3]. Pediatric DLGNT is typically characterized by a diffuse leptomeningeal component, sometimes with a discrete spinal cord lesion and frequently without a clear intraparenchymal component. They often have a low-grade histology, but their clinical behavior is variable and can be aggressive [1, 2, 4, 5]. DLGNTs are characterized by a loss of chromosome arm 1p, sometimes with codeletion of chromosome arm 19q and frequently harbor *KIAA1549::BRAF* fusions [2, 5]. Two methylation subgroups were identified with additionally a gain of 1q in the DLGNT-MC-2 subgroup [1]. Survival rates vary, with case series reporting good overall survival, while in one of the largest cohorts, one-third of the patients succumbed to their tumor [4].

Here, we discuss the extraneural spreading of a DLGNT in a child to the abdominal cavity and the iliac and femoral bones. A patient-derived cell line (PDCL) and patient-derived xenograft (PDX) were developed from the abdominal metastases.

The patient was a 7-year-old girl, previously treated with a ventriculoperitoneal shunt for a Dandy–Walker malformation, who was tentatively diagnosed with a pilocytic astrocytoma with leptomeningeal metastasis. She received treatment according to the SIOP-LGG 2004 protocol, switching vincristine to vinblastine due to neurotoxicity. MRI at the end of induction (week 25) showed partial response and was unchanged until week 85.

Eight months after treatment cessation, imaging indicated progressive disease with new diffuse dural and leptomeningeal metastatic lesions. Cerebrospinal fluid revealed no tumor cells. Biopsy showed diffuse meningeal tumoral infiltration. Immunohistochemistry was positive for GFAP, P53, ATRX, neurofilament, synaptophysin, S100 and MAP2. Ki67/Mib-1 was 80–90%. The tumor had a 1p36.3 loss and a *KIAA1549::BRAF* fusion, without loss of 19q3.3, according to the diagnosis of a DLGNT. Next-generation sequencing showed no mutations in the investigated genes. Methylation array of the tumor corresponded with DLGNT-MC-2 (Fig. 1). She received vinblastine monotherapy, but was clinically progressive. Therefore, she received craniospinal radiotherapy.

**Fig. 1:**
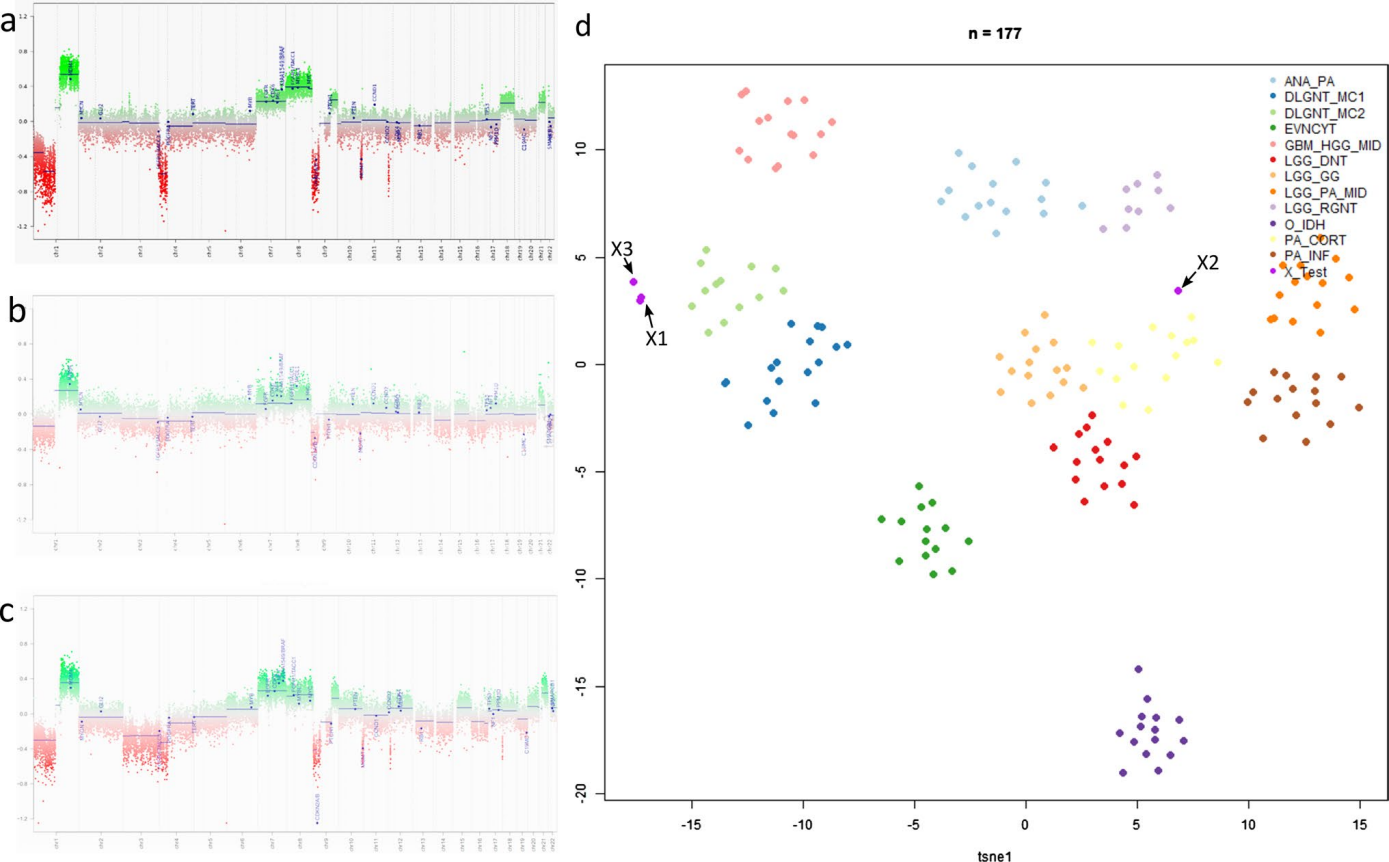
Methylation profiling of the DLGNT and PDCL samples. Copy number alterations of **a** the initial tumor, **b** ascites, **c** PDCL **d** t-SNE plot representing the samples of the initial tumor (X1), ascites (X2) and PDCL (X3) compared to 173 reference samples

MRI at the end of radiotherapy showed partial response and remained stable until 7 months after treatment. Ten months after ending treatment, her neurological symptoms worsened, radiologically corresponding with minor disease progression.

Four months later, she presented with ascites. The peritoneum and omentum were enlarged with small nodular components, suggesting omental metastasis. Iliac and femoral lesions were identified. Histology of the ascites showed malignant, mesothelial and inflammatory cells. The malignant cells were positive for MAP2, synaptophysin and S100, suggestive for a metastatic localization of the DLGNT.

She was again treated with vinblastine, followed by trametinib (MEK-inhibitor). However, the abdominal lesions kept enlarging. Trametinib was stopped due to side effects and progressive disease, and the girl died 3 weeks later. Due to this short treatment period, it is impossible to draw conclusions on the treatment effect on the tumor and its metastases (extended description of the case with images, Online Resources 1 and 2).

Cells isolated from the ascites were cultured and a PDCL was generated, with preservation of the *KIAA1549::BRAF* fusion. Whole-genome sequencing (WGS) revealed loss of chromosomal arm 1p, gain of 1q, and a homozygous *CDKN2A/B* deletion. No *BRAF* V600E mutation, nor mutations in IDH, H3 3A/H3C2 or TERT promoter were observed (materials and methods see Online Resource 3, WGS results see Online resource 4).

The methylation profiles of the tumor and PDCL clustered together with the DLGNT-MC-2 subgroup (Fig. 1). Methylation analysis of the ascites did not show a clear match to the DLGNT-MC-2 subclass, likely due to the altered microenvironmental composition, but showed the same copy number profile as the initial tumor and PDCL. The *KIAA1549::BRAF* fusion (focal 7q34 gain) was detected in all samples. Therefore, these can all be regarded as DLGNT-MC-2. The primary tumor appears to have loss of 9p, but without a focal *CDKN2A/B* deletion. A focal *CDKN2A/B* deletion is clearer in the cells from the ascitic fluid and obvious in the PDCL.

Cytotoxicity assays performed on the PDCL indicated high sensitivity to the treatments the patient received, i.e., vinblastine, trametinib and radiotherapy (Fig. 2), but less to more experimental treatments including the DRD/ClpP modulators ONC206 and ONC212, or the EZH2 inhibitor GSK343.

**Fig. 2:**
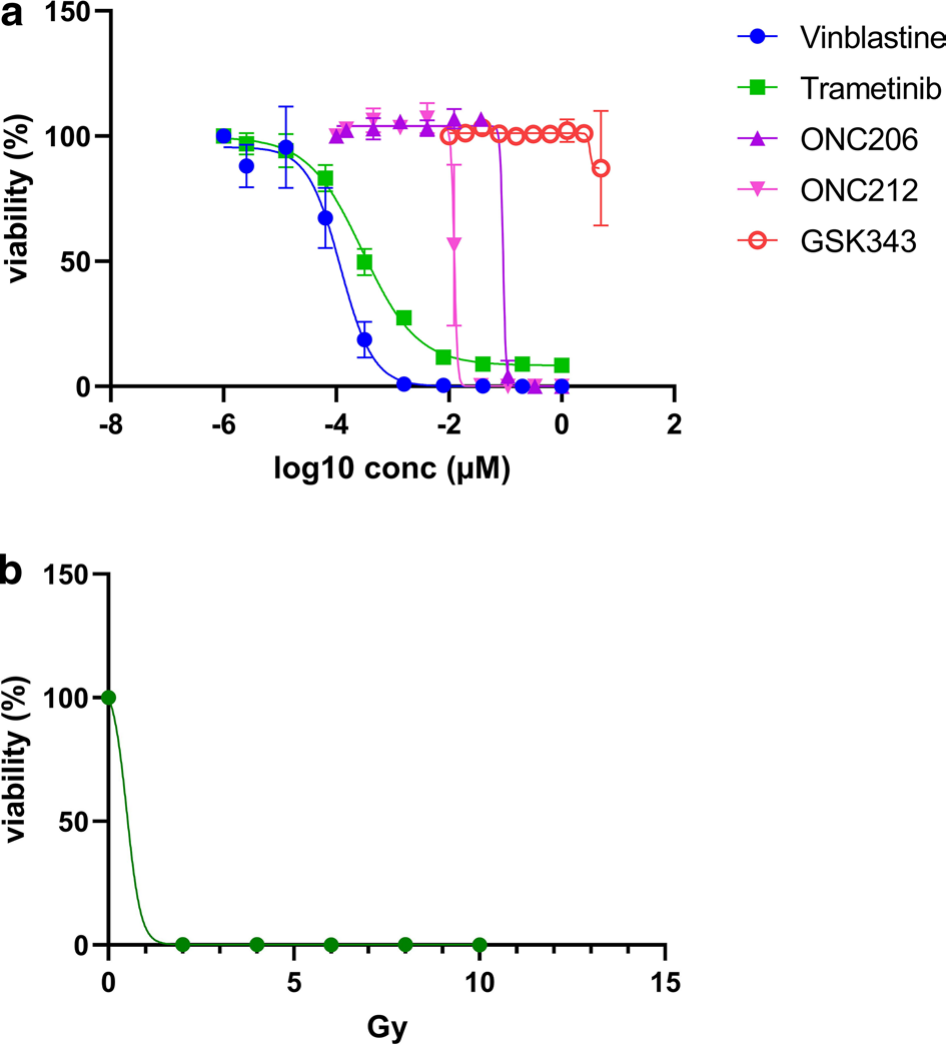
Therapeutic sensitivity profile of the novel DLGNT PDCL. **a** Cytotoxicity profiling using dose–response curves of the PDCL to assess sensitivity towards vinblastine, trametinib, ONC206, ONC212 and GSK343 following a 5-day exposure to each treatment. **b** Cytotoxicity profiling using a dose–response curve following different exposure levels to ionizing radiation. The readout was done after a 6-day incubation period following a single RT exposure

After lentiviral transduction inducing GFP/fLuc positivity, the PDCL was used to generate a PDX model. Of three mice injected, one mouse developed a tumor in the peritoneal cavity (Fig. 3). The cells obtained from the murine tumor were subjected to the same treatments as the PDCL, confirming a similar sensitivity profile (Supplemental Fig. 4, Online Resource 2).

**Fig. 3:**
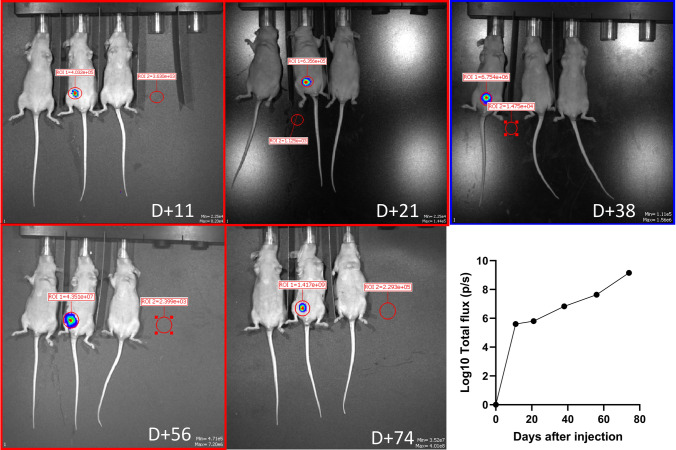
Intraperitoneal engrafting of the DLGNT PDCL to generate a patient-derived mouse model. Bioluminescence imaging of the mice injected with the GFP-fLuc + PDCL (days indicated on image). Graph indicates the evolution of the bioluminescent signal, and thus tumor growth, over time

In conclusion, we presented a case of a DLGNT with a 1p deletion, 1q gain and *KIAA1549::BRAF* fusion displaying an aggressive clinical behavior with metastasis to the peritoneum and bones and described the generation of the corresponding patient-derived models. To date, the possibility of a DLGNT to metastasize to the bones has not been described. The generated models recapitulate the primary molecular characteristics of the original tumor and can be used to study the more aggressive behavior of this disease. However, considering that these were developed from a very malignant tumor, these might not be representative for the initial phase of DLGNT and those with a less malignant phenotype.

## Supplementary Information

Below is the link to the electronic supplementary material.

Online Resource 1: extended description of clinical case (DOCX 18 kb)

Online Resource 2: supplementary figures (DOCX 10641 kb)

Online Resource 3: extended materials and methods (DOCX 23 kb)

Online Resource 4: overview of the WGS data after filtering (XLSX 272 kb)

Online Resource 5: overview of the included genes for qPCR (csv 12 kb)
