# Supplementary material for: Generation of patient-derived models from a metastatic pediatric diffuse leptomeningeal glioneuronal tumor with KIAA1549::BRAF fusion
Source: Acta Neuropathol. 2022 Aug 4;144(4):793–7. doi: 10.1007/s00401-022-02473-w (PMC9468067; doi:10.1007/s00401-022-02473-w)
Supplement: Supplementary file 1 — Online Resource 1: extended description of clinical case (DOCX 18 kb) [file 401_2022_2473_MOESM1_ESM.docx]

**Extended case description**

A 7-year old girl known with a Dandy-Walker variant with enlarged ventricles, presented with a headache and papillary edema. A ventricoloperitoneal shunt (VPS) was therefore placed to treat the increased intracranial pressure.

Magnetic resonance imaging (MRI) six months later showed an intraspinal, intramedullar tumoral lesion, with an irregular ring-shaped contrast enhanced lesion on the T2-T4 level with the impression of swelling of the medulla, with a cystic component further expanding rostral and to a lesser extent caudal (Online resources, supplementary Fig. 1a)*.* Additionally, some T2-hyperintense signals were seen on the rostral and caudal side of this lesion, suggestive of a presyrinx. The cauda equina was enlarged and showed contrast enhancement, together with the leptomeninges thoracolumbal and diffusely pial. 18-F FET PET of the brain showed two foci of enhanced amino-acid metabolism, one in the right mediotemporal lobe and one frontoparietal (Online Resources, supplementary Fig. 1b, Fig. 1c). Both lesions were strongly suspicious of a malign process. Additionally, there was an increased tracer concentration in the low frontal cortex on the midline. Lumbar puncture showed leukorachia with a lymphocytic formula, without signs of malignancy, and a strongly increased protidorachia (25620 mg/L, reference range 150-450 mg/L).

The patient underwent a biopsy of the spinal lesion, and histological examination of the tissue showed cells with round nuclei and a perinuclear halo on a fibrillar background. In between were thin-walled blood vessels, with other zones of more thick-walled hyaline blood vessels (Online Resources, supplementary Fig. 2a-b). Immunohistochemistry was positive for GFAP and ATRX and negative for IDH1. There was no abnormal expression pattern of P53 and the Ki67-Mib1 proliferation index was low. The tentative diagnosis of a pilocytic astrocytoma with leptomeningeal metastasis was made.

She was treated with chemotherapy according to the SIOP-LGG 2004 protocol. After four months, vincristine was switched to vinblastine due to neurotoxicity. MRI at the end of induction (week 25) showed partial response, with a reduction of the contrast enhancement of the lesions. MRI at the end of treatment (week 85) was unchanged.

Eight months after stop of treatment, a novel MRI of the central nervous system indicated progressive disease. New regions of diffuse dural and leptomeningeal metastasis could be seen over the brain and spine. Cerebrospinal fluid (CSF) obtained via a lumbar puncture but also via the VPS was analyzed several times, but revealed no tumor cells. A novel biopsy was therefore performed, which showed diffuse meningeal tumor infiltration. The cells had monotonous nuclei and a pale clear cytoplasm (Online Resources, supplementary Fig. 2c). 22 mitoses/10 high power fields (HPF) were observed. There was no necrosis. Immunohistochemistry showed positivity for GFAP, P53, ATRX, neurofilament, synaptophysin, S100 and MAP2. Ki67/Mib-1 was now 80-90%. The tumor was positive for loss of 1p36.3, negative for loss of 19q3.3 and showed a *KIAA1549::BRAF* fusion, according to the diagnosis of a DLGNT. Next-generation sequencing (NGS) showed no mutations in the investigated genes (*AKT1, ALK, APC, BRAF, CDH1, CTNNB1, EGFR, ERBB2, FBXW7, FGFR2, FOXL2, GNAQ, GNAS, KIT, KRAS, MAP2K1, MET, MSH6, NRAS, PDGFRA, PIK3CA, PTEN, SMAD4, SRC, STK11, TP53*). Methylation array of the tumor (analysis of two separate samples) corresponded with the DLGNT-MC-2 subclass as described before [6].

She was treated with vinblastine monotherapy, but showed no response and was clinically progressive, with an increase in epileptic seizures resistant to anti-epileptic treatment, and progressive somnolence and confusion. Therefore, she received additional craniospinal radiotherapy (36 Gy with a spinal boost to 48.6 Gy and ventricular noduli until 54 Gy).

MRI at the end of radiotherapy showed evidence of partial response with a decrease in the dural and leptomeningeal metastases and remained stable until 7 months after treatment. 10 months after ending treatment, her neurological symptoms worsened, corresponding with minor disease progression on MRI. She was further progressive 2 months later with additional signs of hydrocephalus, for which a revision of the VPS was performed. Due to unsatisfactory relief of the symptoms, the VPS was revised to a ventriculo-cardial shunt.

2 months later, she presented with the sudden development of ascites. The inferior vena cave was compressed, which was treated with the insertion of an endovascular stent. CT-scan of the abdomen confirmed the massive amount of ascites, with signs of increased intra-abdominal pressure. The peritoneum and omentum were enlarged with small nodular components, suggestive for omental metastasis. Additionally, lesions could be seen in both the iliac bones, which were confirmed on MRI of the abdomen and spine. MRI of the abdomen confirmed extensive nodular omental enlargement with diffusion restriction, indicating an omental cake (Online Resources, supplementary Fig. 1d). Furthermore, there was a diffuse contrast enhancement of the peritoneum with some nodularities in the pelvis. The bone marrow in the iliac and femoral bones was inhomogeneous with nodules, again suggestive of metastatic disease (Fig. 1e). Skeletal scintigraphy showed no pathological enhanced tracer activity of these small bone lesions

An abdominal drain was placed, with massive release of fluid. Histological examination of the ascitic fluid showed malignant cells, with irregular, hyperchromatic and slightly enlarged nuclei, next to mesothelial and inflammatory cells. These malignant cells were positive for MAP2, synaptophysin and S100 (Online Resources, supplementary Fig. 2d-f). This was suggestive for a metastatic localization of the known DLGNT. Methylation analysis on the cells obtained from the ascites did not show a clear match to the DLGNT-MC-2 subclass (likely due to the dramatically altered microenvironmental composition) but showed the same copy number profile as the initial tumor (Fig. 1a-c).

She was again started on vinblastine, and was thereafter started on trametinib (MEK-inhibitor). However, despite the start of trametinib, the abdominal lesions kept enlarging. Trametinib was stopped due to its side effects and the progressive disease and the girl died 3 weeks later.
