# Supplementary material for: Generation of patient-derived models from a metastatic pediatric diffuse leptomeningeal glioneuronal tumor with KIAA1549::BRAF fusion
Source: Acta Neuropathol. 2022 Aug 4;144(4):793–7. doi: 10.1007/s00401-022-02473-w (PMC9468067; doi:10.1007/s00401-022-02473-w)
Supplement: Supplementary file 2 — Online Resource 2: supplementary figures (DOCX 10641 kb) [file 401_2022_2473_MOESM2_ESM.docx]

**SUPPLEMENTARY FIGURES**

**Generation of patient-derived models from a metastatic pediatric diffuse leptomeningeal glioneuronal tumor with *KIAA1549::BRAF* fusion.**

Messiaen Julie^1,2^, Claeys Annelies², Shetty Aniket³, Spans Lien^4^, Derweduwe Marleen², Uyttebroeck Anne^1,5^, Depreitere Bart^6,7^, Vanden Bempt Isabelle^4,8^, Sciot Raf^2,9^, Ligon Keith L³, Jones David TW^10,11^, Jacobs Sandra A.^1,5,#^, De Smet Frederik^2,#^

1. Department of Pediatric Hematology and Oncology, University Hospitals Leuven, Leuven, Belgium
2. Translational Cell and Tissue Research, Department of Imaging and Pathology, KU Leuven, Leuven, Belgium
3. Dana Farber Cancer Institute, Department of Pathology, Boston, MA, USA
4. Department of Human Genetics, University Hospitals Leuven, Leuven, Belgium
5. Department of Oncology, KU Leuven, Leuven, Belgium
6. Department of Neurosurgery, University Hospitals Leuven, Leuven, Belgium
7. Research Group Experimental Neurosurgery and Neuroanatomy, Department of Neurosciences, KU Leuven, Leuven, Belgium
8. Department of Human Genetics, KU Leuven, Leuven, Belgium
9. Department of Pathology, University Hospitals Leuven, Leuven, Belgium

(10) Hopp Children´s Cancer Center at the NCT Heidelberg (KiTZ), Heidelberg, Germany

(11) Division of Pediatric Glioma Research, German Cancer Consortium (DKTK), German Cancer Research Center (DKFZ), Heidelberg, Germany

*# shared last authors, corresponding authors*

Corresponding authors:

Prof. Dr. Sandra Jacobs

[Sandra2.jacobs@uzleuven.be](mailto:Sandra2.jacobs@uzleuven.be)

Telephone number:  +32 16 34 17 53

Prof. dr. Frederik De Smet

[Frederik.desmet@kuleuven.be](mailto:Frederik.desmet@kuleuven.be)
Telephone number: +32 16 37 25 75


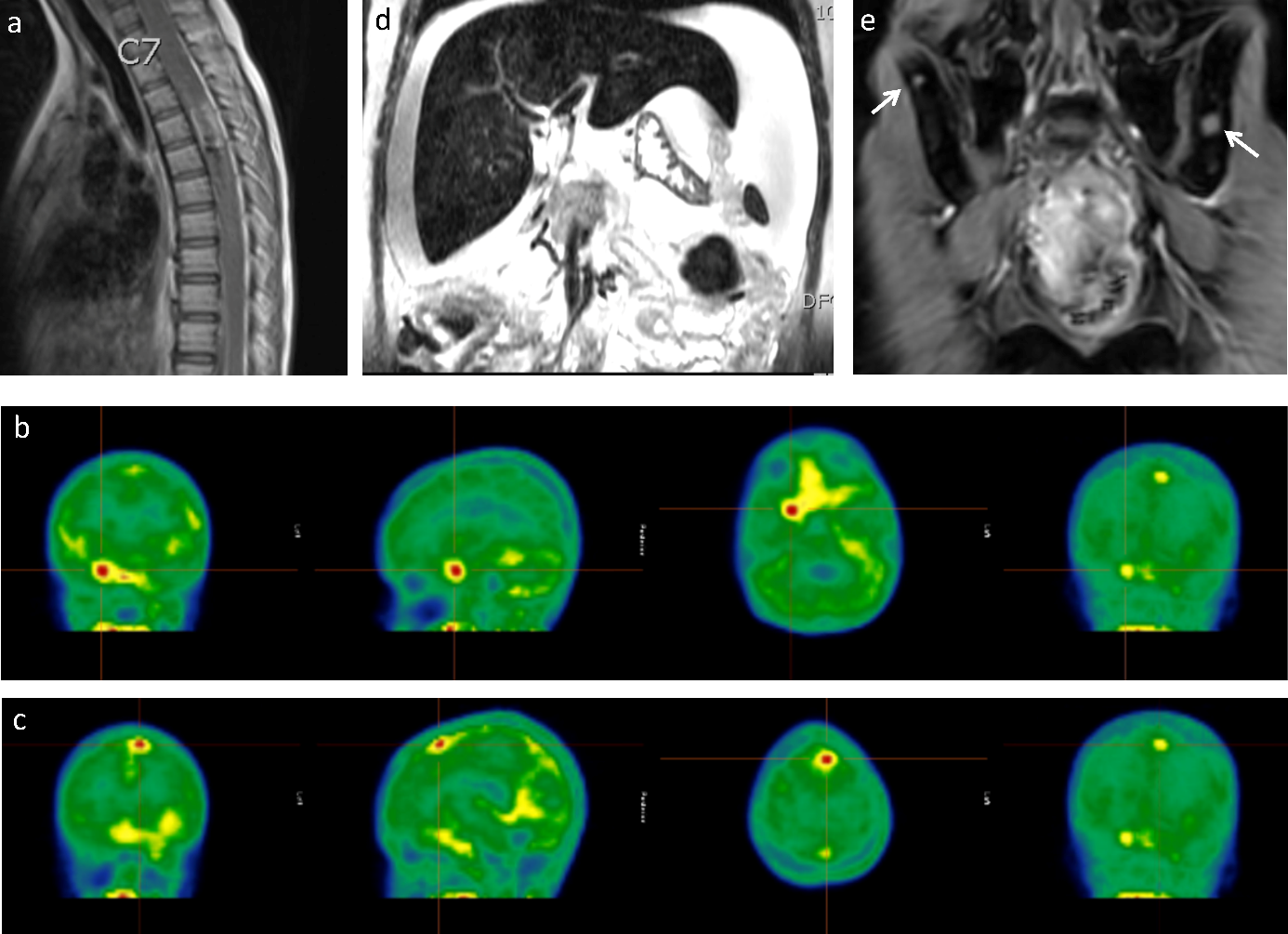


**Supplementary Fig. 1: Imaging results**

1. T1- weighed MRI imaging of the spine illustrating the intraspinal, intramedullar tumoral lesion, with an irregular ring-shaped contrast enhanced lesion on the T2-T4 level with the impression of swelling of the medulla, with a cystic component further expanding rostral and to a lesser extent caudal;
2. 18F FET-PET with focus of strongly enhanced amino acid metabolism mediotemporal right with diffusely enhanced tracer captation at the lower frontal cortex on the midline;
3. 18F FET-PET focus of strongly enhanced amino acid metabolism at the frontoparietal cortex/meninges left;
4. abdominal MRI with T2-weighed image indicating the massive amount of ascitic fluid;
5. T2-weighed MRI image illustrating the bone metastases of the tumor in the iliac bones (indicated with arrows)


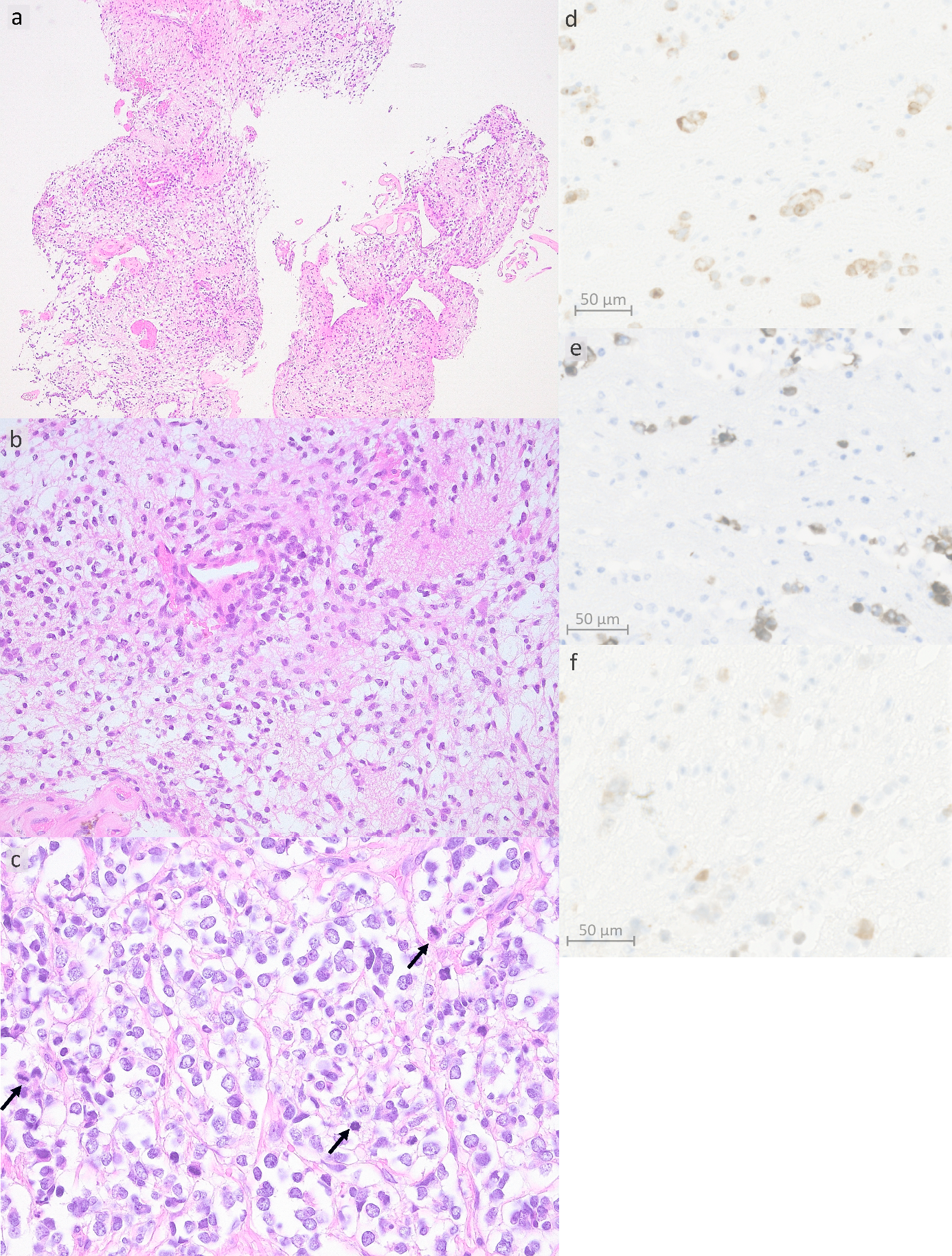


**Supplementary Fig. 2: Histology**

1. Low power view of the primary tumor (H&E staining), showing the moderately increased cellularity and the hypervascular aspect.
2. At high power, the fibrillary background and the perinuclear halos are seen.
3. The recurrent tumor showed an increased cellularity and more atypia, as well as many mitotic figures (arrows).
4. cells of the ascitic fluid stained for synaptophysine (DAB stain);
5. cells of the ascitic fluid stained for MAP2A (DAB stain);
6. cells of the ascitic fluid stained for S100 (DAB stain)


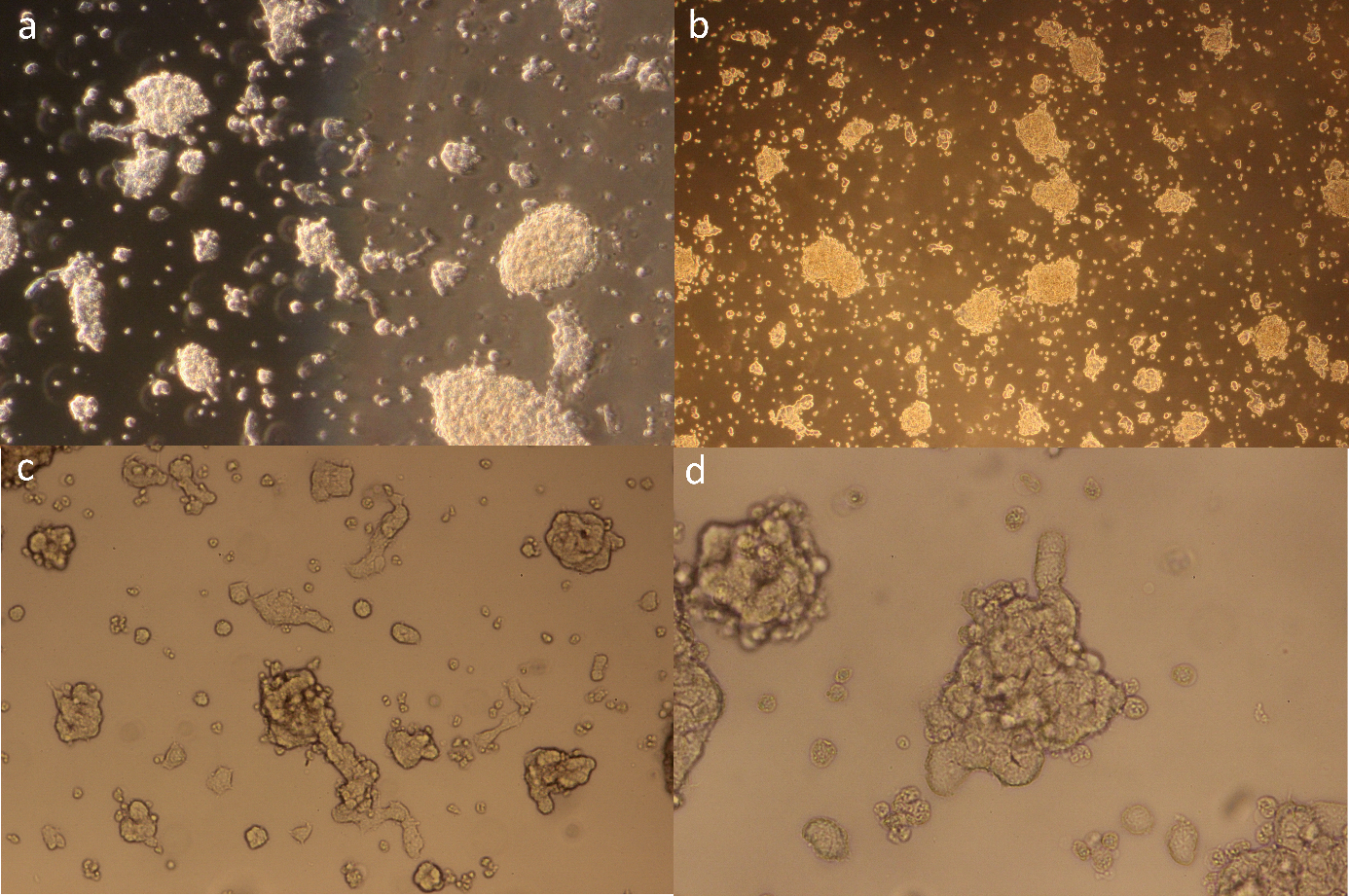


**Supplementary Fig. 3: Microscopic view of the patient-derived cell line**

a) and b) illustrate the cells being partly adhesive, growing in small clumps

c) and d) illustrate the spontaneously delaminating cells forming neurospheres


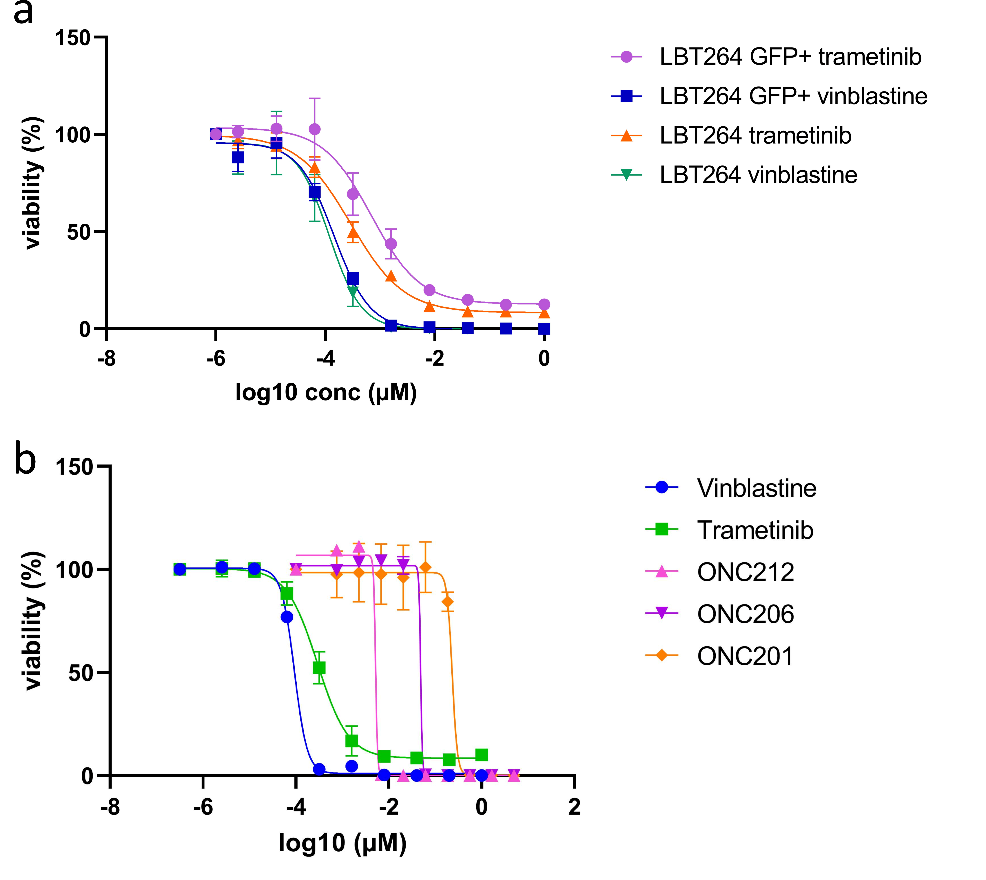


**Supplementary Fig.4: Cytotoxicity assays**

1. dose-response curves for the original PDCL and the GFP-fLuc transduced PDCL after treatment with vinblastine and trametinib after 5 days incubation illustrating similar treatment sensitivities;
2. dose-response curves for the cell-line derived from the PDX tumor for vinblastine, trametinib, ONC201, ONC206 and ONC212 after a 5-day incubation period.
