# Supplementary material for: Generation of patient-derived models from a metastatic pediatric diffuse leptomeningeal glioneuronal tumor with KIAA1549::BRAF fusion
Source: Acta Neuropathol. 2022 Aug 4;144(4):793–7. doi: 10.1007/s00401-022-02473-w (PMC9468067; doi:10.1007/s00401-022-02473-w)
Supplement: Supplementary file 3 — Online Resource 3: extended materials and methods (DOCX 23 kb) [file 401_2022_2473_MOESM3_ESM.docx]

**Generation of patient-derived models from a metastatic pediatric diffuse leptomeningeal glioneuronal tumor with *KIAA1549::BRAF* fusion.**

Messiaen Julie^1,2^, Claeys Annelies², Shetty Aniket³, Spans Lien^4^, Derweduwe Marleen², Uyttebroeck Anne^1,5^, Depreitere Bart^6,7^, Vanden Bempt Isabelle^4,8^, Sciot Raf^2,9^, Ligon Keith L³, Jones David TW^10,11^, Jacobs Sandra A.^1,5,#^, De Smet Frederik^2,#^

1. Department of Pediatric Hematology and Oncology, University Hospitals Leuven, Leuven, Belgium
2. Translational Cell and Tissue Research, Department of Imaging and Pathology, KU Leuven, Leuven, Belgium
3. Dana Farber Cancer Institute, Department of Pathology, Boston, MA, USA
4. Department of Human Genetics, University Hospitals Leuven, Leuven, Belgium
5. Department of Oncology, KU Leuven, Leuven, Belgium
6. Department of Neurosurgery, University Hospitals Leuven, Leuven, Belgium
7. Research Group Experimental Neurosurgery and Neuroanatomy, Department of Neurosciences, KU Leuven, Leuven, Belgium
8. Department of Human Genetics, KU Leuven, Leuven, Belgium
9. Department of Pathology, University Hospitals Leuven, Leuven, Belgium

(10) Hopp Children´s Cancer Center at the NCT Heidelberg (KiTZ), Heidelberg, Germany

(11) Division of Pediatric Glioma Research, German Cancer Consortium (DKTK), German Cancer Research Center (DKFZ), Heidelberg, Germany

*# shared last authors, corresponding authors*

Corresponding authors:

Prof. Dr. Sandra Jacobs

[Sandra2.jacobs@uzleuven.be](mailto:Sandra2.jacobs@uzleuven.be)

Telephone number:  +32 16 34 17 53

Prof. dr. Frederik De Smet

[Frederik.desmet@kuleuven.be](mailto:Frederik.desmet@kuleuven.be)
Telephone number: +32 16 37 25 75

**MATERIALS AND METHODS**

**Patient selection and sample accrual**

The study was performed at the University Hospitals Leuven (Belgium) after approval of the Local Institutional Review Board (study number s59804). Informed consents were signed by the parent. The study was performed in accordance with the Declaration of Helsinki and its later amendments. A sample of the abdominal fluid was obtained from the patient via the already implanted abdominal drain. No additional invasive procedures were performed to obtain the sample. Clinical information was retrieved from the patient records.

**Immunohistochemical staining**

Routine immunohistochemical staining and evaluations were performed as part of the diagnostic track in the department of Pathology of the University Hospitals Leuven (Belgium). Histopathological examination of the primary tumor was performed on formalin-fixed paraffin embedded tissue (FFPE). Routinely, 5 µm sections were used for hematoxylin and eosin (H&E) staining. Immunohistochemistry was performed using the avidin-biotin-peroxidase complex method. The following antibodies where used for immunohistochemistry: ATRX (Sigma Aldrich, RTU, HPA001906); GFAP (Dako, RTU, GA524); Ki-67 (Dako, RTU, IR626); MAP2 (Sigma Aldrich, RTU, M9942); NF (Dako, RTU, GA607); P53 (Dako, RTU, IR616); S100 (Dako, RTU, IR504); Synaptophysin (Dako, RTU, GA660).

**Cell line generation**

A sample of the abdominal fluid was obtained from the abdominal drain of the patient. 10 ml of ascitic fluid was centrifuged at 300g for 5 minutes (Hettich centrifuge).

The cells were grown in Neurocult NS-A Basal medium (human) + supplements (human) (Stem cell technologies), with added EGF and FGF2 (Stem Cell Technologies, 78006.2 and 78003.2 respectively) to a concentration of 20 ng/ml. Heparin (Stem Cell technologies, #07980) was added to a final concentration of 0.0002%. Antibiotic-antimycotic (Life technologies, 15240-062) was added to a concentration of 1%.

Before seeding the cells, a T25 flask (Corning, 430639) was coated with laminin (Sigma-Merck Life Science, L2020-1MG) diluted in phosphate-buffered saline (PBS; Gibco, 14190250) to a concentration of 25 µg/ml. Laminin was incubated for at least one hour at 37°C. The remnant laminin solution was removed before seeding of the cells. The cells were kept in an incubator at 37°C with humidity and gas control and an atmosphere of 5% CO2 in air. Medium was changed 2x/week and the cells were split when reaching 80% confluency. Cells were removed from the flask using a cell scraper (Sarstedt).

The PDCL was regarded as established after passing passage 3.

The pictures of the PDCL were made using a camera attached to an inverted microscope.

**DNA and RNA isolation**

RNA was extracted from the cell line at passage 3 using the RNeasy mini kit (Qiagen) according to the manufacturer’s instructions. Genomic DNA was extracted from the cell line at passage 7 using the DNeasy Blood & Tissue Kit (Qiagen) and was used for sequencing and methylation analysis. DNA concentration was measured using the Quantus fluorometer (Promega).

**Gene expression analysis by quantitative real-time PCR**

cDNA was made with the GoScript reverse transcription system (Promega), starting from 1 µg of RNA. The expression profile of 64 genes was measured by quantitative PCR using PrimeTime qPCR Probe Assay (IDT) and GoTaq Probe qPCR master Mix (Promega) and run on a CFX96 Touch Deep Well Real-Time PCR System (Bio-Rad) (see Online Resource 5 for the included qPCR primers). The following PCR program was used: 95°C for 20 seconds, 95°C for 5 seconds, 60°C for 25 seconds with 40 cycles of step 2 and step 3. Data analysis was performed as previously described in [1] using R-Studio v1.2.1335.

**Archer DX**

RNA was provided for analysis using the Archer Fusionplex CTL (Invitae), according to the manufacturer’s methods. The analysis was performed by the Center of Human Genetics, University Hospitals Leuven according to their standard clinical procedures. The Archer Analysis software version 6.0.4. was used for data-analysis.

**Whole genome sequencing**

Extracted DNA of the PDCL was sent to Dante Labs (<https://www.dantelabs.com/>) for whole genome sequencing (WGS) analysis (material and data transfer agreement in place) (coverage 41.6X). The VCF’s were generated by Dante Labs using the standard DRAGEN pipeline (Illumina). The VCF’s were analyzed by S.A. To annotate variants with additional information, the VCF file was annotated with additional information in order to get a sense of variants impact on a phenotype using Ensembl Variant Effect Predictor (VEP) which was further converted into Mutation Annotation Format (MAF) file using vcf2maf tool. The resulting MAF file was then annotated using OncoKB database. The data was filtered to include only the non synonymous variants, then were further filtered using the following approach: the COSMIC counts and variant allele frequencies were added, after which variants that did not appear in COSMIC or in OncoKB or that were not annotated by mutect filter as “pass” were removed. Additionally, variants were filtered out using the gnomAD frequency, but cases occurring at least twice in COSMIC were rescued. Furthermore, low variant allele frequencies were also filtered out, but again, cases which occurred at least twice in COSMIC were rescued. As a last step, silent mutations were filtered out. Mutations with a variant allele frequency of >10% were investigated. Mutations that were detected as possibly harmful by SIFT, ClinVAR, COSMIC or OncoKB were further investigated. These were searched in Pubmed and if these were described in literature as being involved in malignancy, these were included.

**Cytotoxic assays**

Before seeding, the 96-well plates (Thermo Scientific, Nunc^TM^ Edge 2.0, 735-0330) were coated with laminin diluted in PBS for 1-3 hours. 10 wells per row were coated and drug experiments were performed in duplicate or triplicate. The outer rows of the plates were not used to avoid effect of evaporation on the results and were filled with 200 µL PBS.

The cells were removed from the flask using a cell scraper and were dissociated to a single cell suspension using Stempro accutase (Gibco). The cells were counted using Cell counting slides (Bio-Rad, 1450011) in the TC20 Automated Cell Counter (Bio-Rad, #145-0101) with a trypan blue (Bio-Rad, 1450013) staining. Cells were seeded at a concentration of 10 000 live cells per well in a 96-well plate.

Compounds were added in a dilution series the day after the seeding of the cells. Compounds were diluted in dimethyl sulfoxide (DMSO; Merck Life Science, D2650) and one well was treated with DMSO as a control. The read-out of the plates was performed using CellTiter-Go Luminescent Cell Viability Assay (Promega) according to the manufacturer’s instructions. The read-out was performed in the Spectramax iD3. Data-analysis was done using GraphPad Prism (version 8.4.3).

The following components were used for the cytotoxic assays: trametinib (Selleckchem, GSK1120212), vinblastine sulfate (Sellekchem, NSC 49842), GSK343 (BD Biosciences, S7164), ONC201 (MedChemExpress, HY-15615A), ONC206 (MedChemExpress, HY-135147), ONC212 (MedChemExpress, HY-111343).

Treatments with radiotherapy were performed using the RS-2000 Biological irradiator (Rad Source Technologies), generating 4 Gy/minute. Cells were treated with fractions of 2 Gy, 4 Gy, 6 Gy, 8 Gy and 10 Gy and were thus irradiated for respectively 30, 60, 90, 120 and 150 seconds. The read-out of this experiment was performed after 6 days, in the same manner as described above.

**Lentiviral transduction**

GFP-fLuc lentivirus was obtained from the Leuven Viral Vector Core (KU Leuven). Cells were dissociated using Stempro accutase (Gibco) and were plated in a 6-well plate with a density of 1 000 000 cells/well. Lentivirus was added 4 hours after seeding in a serial dilution. 24 hours later, the excess virus was replaced by fresh medium. We continued with the dilutions that yielded 30 to 60% of GFP positive cells.

**Sorting of GFP-fLuc+ cells**

Sorting of the lentiviral transduced cells was performed on a Sony MA900 cell sorter after being dissociated with Stempro accutase (Gibco). The dissociated cells were kept in sorting buffer containing PBS, 2% fetal bovine serum (FBS; Thermo Fisher Scientific,
10500-064) and 2 mM EDTA (Thermo Fisher Scientific, 15575020). After sorting, the cells were plated in a T25 flask, coated with laminin and were grown under the same conditions as previously described.

**PDX generation**

Sorted GFP-fLuc+ cells were used for injection into mice. GFP-fLuc positivity of the cells before implantation was evaluated with a fluorescence microscope. The cells were prepared for injection by dissociation into a single cell suspension using a 2-minute incubation step with Stempro accutase (Gibco).

Xenograft development was performed in 6 weeks old immunocompromised female NMRI mice (nu/nu, Janvier, France). No analgesia was administered since the mice were immediately injected intraperitoneally with the tumor cells. A total of 2 million cells in 200µL were injected per mice.

The mice were housed in a temperature- and light-controlled environment with an alternating 12-hour light and dark cycle. Tumor growth and evolution was monitored at different time points using bioluminescent imaging (BLI; PerkinElmer IVIS spectrum In Vivo Imaging System). For imaging, the mice were anesthetized using isoflurane inhalation in a covered box. Imaging was performed 10 minutes after intraperitoneal injection of luciferin in aqueous buffer (Promega, E1605). Image analysis was performed using the Living Image software (version 4.5.4).

The general status of the mice was frequently observed. The mice were sacrificed by decapitation. Immediately thereafter, the tumor was removed.

During the entire experiment, the laws regarding experimentation on animals were followed.

**PDX tumors dissociation**

The tumor of the PDX was dissociated using the Brain Tumor Dissociation Kit (Miltenyi Biotec) according to the manufacturer’s instructions. The cells were seeded as described previously in a T25 flask with DMEM-F12 medium, supplemented with EGF, bFGF, heparin and antibiotic-antimycotic in the same concentrations as described previously. Review of the cells under a fluorescent microscope confirmed fluorescence of the cells. The cells were handled as described previously. The generated cells from this tumor were subjected to cytotoxic assays, executed as described above.

**DNA methylation assay**

Extracted genomic DNA from the primary tumor, the cells from the ascitic fluid and the PDCL was used to perform whole genome methylation analysis. The analysis was performed by the Centre of Human Genetics of the University Hospitals Leuven, using the Infinium MethylationEPIC Beadchip (Illumina, 850k arrays) according to the manufacturer’s instructions. The data was analyzed by DTWJ using the DKFZ Molecular Neuropathology (MNP) Classifier version 12.

**REFERENCES**

[1] J. Vandesompele, K. De Preter, F. Pattyn, et al., Accurate normalization of real-time quantitative RT-PCR data by geometric averaging of multiple internal control genes, Genome Biol. 3 (2002). https://doi.org/10.1186/GB-2002-3-7-RESEARCH0034.
